# *Toxoplasma gondii* Syntaxin 6 Is Required for Vesicular Transport Between Endosomal-Like Compartments and the Golgi Complex

**DOI:** 10.1111/tra.12102

**Published:** 2013-09-12

**Authors:** Allison J Jackson, Caroline Clucas, Nicola J Mamczur, David J Ferguson, Markus Meissner

**Affiliations:** 1 Wellcome Trust Centre for Molecular Parasitology, Institute of Infection, Immunity and Inflammation, College of Medical, Veterinary and Life Sciences, The University of GlasgowGlasgow, G12 8TA, UK; 2 Nuffield Department of Clinical Laboratory Science, University of OxfordOxford, OX3 9DU, UK

**Keywords:** conditional mutant, endosomes, Golgi, Syntaxin, TGN, *Toxoplasma gondii*, vesicular traffic

## Abstract

Apicomplexans are obligate intracellular parasites that invade the host cell in an active process that relies on unique secretory organelles (micronemes, rhoptries and dense granules) localized at the apical tip of these highly polarized eukaryotes. In order for the contents of these specialized organelles to reach their final destination, these proteins are sorted post-Golgi and it has been speculated that they pass through endosomal-like compartments (ELCs), where they undergo maturation. Here, we characterize a *Toxoplasma gondii* homologue of Syntaxin 6 (TgStx6), a well-established marker for the early endosomes and *trans* Golgi network (TGN) in diverse eukaryotes. Indeed, TgStx6 appears to have a role in the retrograde transport between ELCs, the TGN and the Golgi, because overexpression of TgStx6 results in the development of abnormally shaped parasites with expanded ELCs, a fragmented Golgi and a defect in inner membrane complex maturation. Interestingly, other organelles such as the micronemes, rhoptries and the apicoplast are not affected, establishing the TGN as a major sorting compartment where several transport pathways intersect. It therefore appears that *Toxoplasma* has retained a plant-like secretory pathway.

Apicomplexan parasites such as *Toxoplasma gondii* or *Plasmodium spp*. cause some of the most devastating diseases in humans and domestic animals. Most of these obligate intracellular parasites establish a niche inside their host cell and reside within a parasitophorous vacuole (PV) that is established during invasion (1). Furthermore, these parasites extensively remodel their host cell, most impressively observed in case of infected erythrocytes by *Plasmodium* (2). While the mechanisms and virulence factors involved in host-cell modifications vary within the apicomplexans (3), these proteins are sorted within the secretory system of the parasite and secreted in either a constitutive or regulated manner (4). Curiously, apicomplexans possess unique secretory organelles that fulfil key functions during invasion (micronemes and rhoptries) and host-cell modulation (rhoptries and dense granules). During invasion these organelles are secreted in a stepwise manner, deploying a whole arsenal of important virulence factors (5).

The apicomplexan secretory system is highly polarized, consisting of the endoplasmic reticulum (ER), a single Golgi stack (6) and ill-defined post-Golgi compartments, which we refer to as the endosomal-like compartment (ELC) owing to their colocalization with typical endosomal markers, such as Rab5-GTPases or Cathepsin-like proteins (7–10). Some micronemal and rhoptry proteins appear to mature by proteolytic processing at the ELC (7,^11^,12). Recently, a plant-like vacuole (PLV or VAC) has been described as an ELC-associated compartment (11,13). The micronemes and rhoptries can be identified at the apical pole of the parasite, whereas dense granules can be found more evenly distributed throughout the parasite (4). Secretory proteins cotranslationally enter the ER via the signal recognition particle pathway (14). Subsequently, proteins are trafficked to their final destination and some sorting signals have been identified for the targeting to the micronemes and rhoptries. Interestingly, Rab5-GTPases have been shown to play a crucial role in the transport of rhoptry proteins and a subset of micronemal proteins (8), indicating a modulation of the endocytic trafficking pathway in apicomplexans.

Endosomes are sorting organelles where secretory and endocytic traffic intersect in diverse eukaryotic phyla (15). They accept and dispatch vesicles from the *trans* Golgi network (TGN), the plasma membrane and the lysosome. In opisthokonts (a eukaryotic superclade comprising Metazoa and a few unicellular lineages, including Fungi) the general pathway can be described as a stepwise transport from the early endosomes (EEs) to late endosomes (LEs) to the lysosome (16); however, EEs have so far not been identified in plants (17). Instead, an elaborate TGN directly receives endocytic vesicles from the plasma membrane and the Golgi. Plant cells contain multiple vesicular bodies that correspond to LEs of animal cells (18). Much of our knowledge on the general organization of endosomes in eukaryotes has been derived from forward genetic screens performed in yeast to identify transport defects of carboxypeptidase Y to the yeast vacuole, which is analogous to the lysosome. To date, more than 70 different vacuolar protein sorting (VPS) mutants have been identified (19) and grouped into six classes (A–F). Subsequent analysis demonstrated that proteins from one class are functionally linked and often form a complex. To date, few homologues of *vps* genes have been studied in detail in *T. gondii*. While the homologue of VPS10 (or sortilin) has been described as the sorting receptor for the transport of micronemal and rhoptry proteins from the Golgi to ELC (10), the dynamin-related protein B, a homologue of VPS1, is essential for biogenesis of micronemes and rhoptries (20). Finally, the Rab5-GTPases Rab5A and Rab5B are homologues of yeast VPS21 and are essential for the transport of rhoptry and microneme proteins (8). Interestingly, in all cases, micronemal and rhoptry proteins are secreted constitutively into the PV, just as is the case for carboxypeptidase Y in the corresponding yeast mutants (21). This indicates remodelling of the pathways involved in endocytosis and transport to lysosomes to facilitate the specific transport of micronemal and rhoptry proteins to their respective organelles, which might have evolved from lysosomes (22).

To further our studies on vesicular transport in apicomplexan parasites, we have begun to characterize SNARE (soluble NSF receptor) gene families that may be involved in traffic between ELCs and the Golgi. SNARE proteins are involved in membrane fusion and act in concert with other SNAREs to deliver membrane-bound cargo around the cell. Various SNAREs have been well characterized in plants and yeast, but SNAREs have not yet been functionally analysed in apicomplexans.

Among the 23 SNARE-like proteins identified in the genome of *T. gondii* we detected a highly conserved homologue of Syntaxin 6 (Stx6), also known as Syp61 in plants. Stx6 has been well described in yeast and is localized at the TGN and EEs (23,24). Several roles have been assigned to Stx6 (25), including retrograde transport from EEs to the TGN (26,27), caveolin-dependent endocytosis (28) and regulation of constitutive secretion (25). Similarly, in plants Syp61 has been implicated in promiscuous roles at the TGN, such as retrograde traffic from the prevacuolar compartment to the TGN (29), and a role in exocytosis and transport of material to the cell wall (30).

Here, we present the first analysis of an apicomplexan SNARE, TgStx6, and demonstrate that it localizes to the TGN and the surface of the parasite, and between the Golgi and ELC. Overexpression of TgStx6 is deleterious and blocks transport from the ELC to the Golgi of the parasite. In addition, we found a defect of the formation of the inner membrane complex (IMC) during parasite replication, while the apical organelles remained intact. Together, our data suggest that similar to plants, the apicomplexan TGN is a major sorting organelle, where the transport routes for constitutive secretion, the IMC and to the unique secretory organelles (micronemes and rhoptries) diverge.

## Results

### Syntaxin 6 is highly conserved in apicomplexan parasites

**Figure 1:**
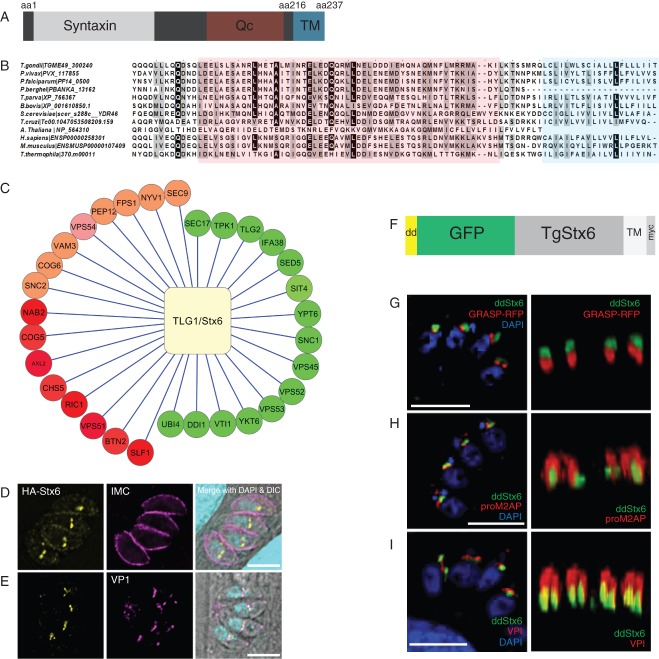
TgStx6 is conserved and localizes next to the ELC and the Golgi. A) Domain overview of the *T. gondii* Stx6 protein (TGME49_300240). TgStx6 has a C-terminal TM domain, a Qc-SNARE domain and a Syntaxin domain. B) Alignment of the C-terminal region of several eukaryotic Stx6 homologues. The Qc-SNARE domain is shaded red. The TM domain is shaded blue. Identical residues are indicated in black; those with 75% or greater identity are shaded grey. C) Repertoire of putative TLG1/TgStx6 interaction partners. Those conserved in *T. gondii* and yeast are shaded green; those conserved in yeast and other eukaryotes are shaded orange; and those unique to yeast are coloured red. D) Epifluorescence images showing the localization of HAStx6 (yellow) in pstx6-HAStx6 parasites in relation to the IMC (magenta) and nucleus (cyan); E) the ELC using anti-VPI antibodies (magenta). HAStx6 resides postnuclear and partially colocalizes with the ELC. F) Schematic of the ddTgStx6 construct used in this study. TgStx6 (grey) is N-terminally tagged with the destabilization domain (yellow), myc tag (light grey) and GFP (green). G) Epifluorescence analysis and a 3D reconstruction of ddStx6 parasites transiently transfected with the Golgi marker GRASP-RFP. TgStx6 is positioned above the Golgi. H) Epifluorescence analysis and a 3D reconstruction of ddStx6 parasites probed with proM2AP antibodies to stain the ELC. ddStx6 sometimes colocalizes with proM2AP. I) Epifluorescence analysis and a 3D reconstruction of ddStx6 parasites stained with VP1 antibodies to mark the ELC. ddStx6 sometimes colocalizes with VP1. The ELC lies above TgStx6. Scale bars are 5 μM.

In order to define post-Golgi trafficking steps in *T. gondii*, we searched for SNAREs that may be implicated in endosomal transport and identified the apicomplexan homologue of Syntaxin 6 (TgStx6, TGME49_300240) as an ideal candidate. First, it appears to be conserved in all apicomplexan parasites, indicating a critical role in vesicular transport. Furthermore, although its function is slightly different in yeast and plants, it is known to localize to the TGN and EEs (29,31); neither of these organelles have been convincingly demonstrated in *Toxoplasma*. An alignment of different orthologues demonstrates that TgStx6 has a conserved C-terminal transmembrane (TM) domain, which is necessary for insertion into vesicle membranes, and an alpha-helix-containing Qc-SNARE domain that permits interaction with other SNAREs [Figure 1A,B (32)]. TgStx6 also contains an N-terminal regulatory domain known as a Syntaxin or Habc domain. We generated a putative interaction network of TgStx6, looking for homologues for all characterized interactions with yeast Tlg1p in the *T. gondii* genome (Figure 1C and Table S1, Supporting Information). Interestingly, the repertoire of putative interaction partners appears to be highly reduced in apicomplexan parasites. Notably, components involved in retrograde traffic from the endosome to the Golgi, such as components of the GARP complex (33), are highly conserved between yeast and apicomplexan parasites (Figure 1C and Table S1).

### TgStx6 localizes between the Golgi and ELC

To analyse the localization of TgStx6 we initially attempted to express Stx6 fusion proteins with either GFP or HA (HA-Stx6 and GFP-Stx6). In transient transfection assays, we found the respective fusion proteins localized close to the nucleus and a weak signal was observed at the periphery of the parasite (Figure S1A). However, we were unable to generate transgenic parasites that constitutively express HA-Stx6 or GFP-Stx6, indicating that overexpression of TgStx6 is not tolerated by the parasite. To visualize the localization of the endogenous protein, we attempted to tag endogenous *tgstx6*. However, none of the constructs used allowed C-terminal tagging, probably owing to interference with the TM domain of TgStx6, which is required for inserting the SNARE into its target membrane. We also attempted to generate peptide antibodies to TgStx6, but failed to detect the endogenous protein (data not shown). Finally, we attempted to generate an inducible knockout of a *ha*-tagged *tgstx6*. Therefore, we introduced a *ha-tgstx6* flanked by loxP sites in the parental parasite strain *ku80::diCre*, which contains dimerizable Cre-recombinase (34). Unfortunately, we were unable to exchange this construct for endogenous *tgstx6* (data not shown), indicating that even slight alterations of the gene expression or tagging of the protein is not tolerated by the parasite. However, in the resulting transgenic parasites, *ha*-tagged *tgstx6* is expressed under the control of its native promoter (pstx6-HAStx6), and therefore likely to reflect endogenous TgStx6 expression. Immunofluorescence analysis (IFA) showed that HA-TgStx6 displayed a postnuclear localization in the pstx6-HAStx6 parasites (Figure 1D) and partially colocalized with the ELC marker VP1 (Figure 1E). pstx6-HAStx6 can also be seen at times at the periphery of the parasite, colocalizing with the IMC protein 1 (IMC1, Figure 1D). This localization is consistent with that observed in the transient assays using the tagged TgStx6 (Figure S1A).

To define the precise nature of the Stx6 compartment, we analysed its subcellular localization in comparison to other markers of the endocytic/exocytic system. As we were unable to get stable expression of the HA-and GFP-Stx6 constructs we employed the ddFKBP system that allows rapid and tuneable regulation of proteins of interest (35). We N-terminally tagged TgStx6 with ddFKBP-myc-GFP and succeeded in generating transgenic parasites [ddFKBP-myc-GFP-TgStx6 (referred to as ddStx6; Figure 1F)].

Parasites expressing ddStx6 showed a distinct structure anterior to the nucleus, as previously observed for the pstx6-HAStx6 parasites (Figure 1D,E). However, when we performed colocalization studies using parasites coexpressing ddStx6 and the Golgi marker GRASP-RFP (36), we found that the TgStx6 compartment was juxtaposed apically to the Golgi (Figure 1G). In three-dimensional (3D) reconstructions it appeared that the TgStx6 compartment is next to the Golgi with no significant overlap. In contrast, in immunolocalization studies using antibodies against different subdomains of the ELC, proM2AP and VP1 (11,13), we consistently found TgStx6 aligned to proM2AP and VP1 signals with a clear, although only partial colocalization (Figure 1H,I). These localizations were consistent with what we observed with the pstx6-HAStx6 parasites (Figure 1D,E).

**Figure 4:**
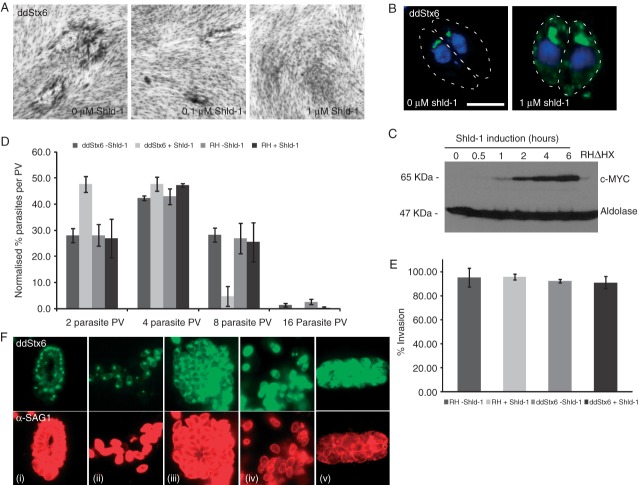
TgStx6 is essential for parasite survival: phenotypic characterization of ddStx6 parasite mutants. A) Growth analysis of the ddStx6 parasites grown on HFF cells for 5 days. In the ddStx6-expressing parasites, plaque size is reduced with increasing Shld-1 concentrations. B) Epifluorescence analysis of stable clones containing ddStx6 (−/+Shld-1). ddStx6 parasites have some background expression, with an expanded and perturbed staining, and some plasma membrane localization upon the addition of Shld-1. Scale bar is 5 μM. C) Western blot analysis of Shld-1 regulation of ddStx6 showing the induction of ddStx6 expression at various time points indicated after the addition of Shld-1 using an anti-myc antibody. RHΔHX parasites were used as a control and were induced for 6 h. Aldolase was used as a loading control. D) Replication is slightly delayed in ddStx6-expressing parasites. The parasites were treated with (+/−) Shld-1 and allowed to invade HFF cells. After 24 h the parasites were fixed and stained with α-aldolase. The percentage of PV containing either 2, 4, 8 or 16 parasites was counted. The results shown are an average of three independent experiments (*n*_ave_ = 100). Error bars are the SEM. E) Invasion ability is not compromised upon ddStx6 expression. Lysed parasites were induced with (+/–) Shld-1 for 6 h before invading HFF monolayers for 1 h. IFA was performed, and the number of invaded and extracellular, attached parasites was counted. The results shown are an average of two independent experiments (*n*_ave_ = 192). There is no difference in invasion ability between RHΔHX parasites and of ddStx6 parasite mutants in the presence or absence of Shld-1. Error bars are 95% confidence intervals. F) Egress of the host cell is not blocked upon overexpression of TgStx6. (i) Untreated ddStx6 parasites without treatment; (ii) untreated ddStx6 parasites induced with 1 μM calcium ionophore; (iii) ddStx6 parasites treated with Shld-1 for 6 h without egress induction; (iv) ddStx6 parasites treated with Shld-1 for 6 h and induced with 1 μM calcium ionophore; (v) ddStx6 parasites treated with Shld-1 for 12 h and induced with 1 μM calcium ionophore. Parasites were stained with α-SAG1. This result was observed on three independent occasions.

**Figure 5:**
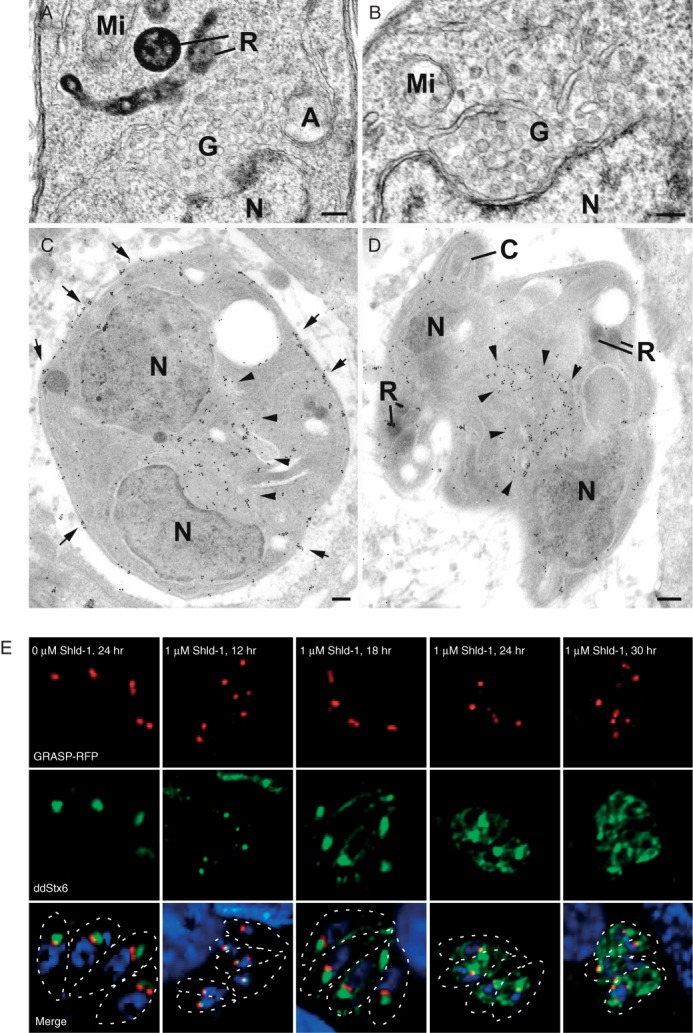
Overexpression of TgStx6 causes Golgi fragmentation. Routine EM (A, B) and immuno-EM (C, D) images of ddStx6 parasites (grown in the presence of 1 μM Shld-1 and overexpressing ddStx6) labelled with anti-GFP and were visualized with 10-nm gold particles. Scale bars indicate 100 nm. A, B) Sections through the Golgi region (G) located just anterior to the nucleus (N). Note the absence of the stacked membranes being replaced by numerous vesicular structures. A, apicoplast; R, rhoptry; Mi, mitochondrion. C, D) Sections through parasites showing abnormal development in which there are large numbers of gold particles associated with the central perinuclear region (arrowheads) and the IMC (arrowheads) of the mother cell. N, nucleus; C, conoid; R, rhoptry. E) ddStx6 parasites transiently transfected with RFP-GRASP (−/+Shld-1) and fixed at 12, 18, 24 and 30 h post-transfection. GRASP localization does not change upon TgStx6 overexpression.

Together, these results indicate that TgStx6 localizes to a post-Golgi compartment close, but distinct, to the ELC. Interestingly, both the pstx6-HAStx6 parasites and overexpression of ddStx6 result in a partial surface localization (Figures 1D, 4B and 5C). Therefore, we speculate that TgStx6 plays a role during the vesicular transport from the TgStx6 compartment to the ELC and possibly to the parasite surface or IMC.

### Segregation of the TgStx6 compartment is tightly linked to the Golgi and ELC

**Figure 2:**
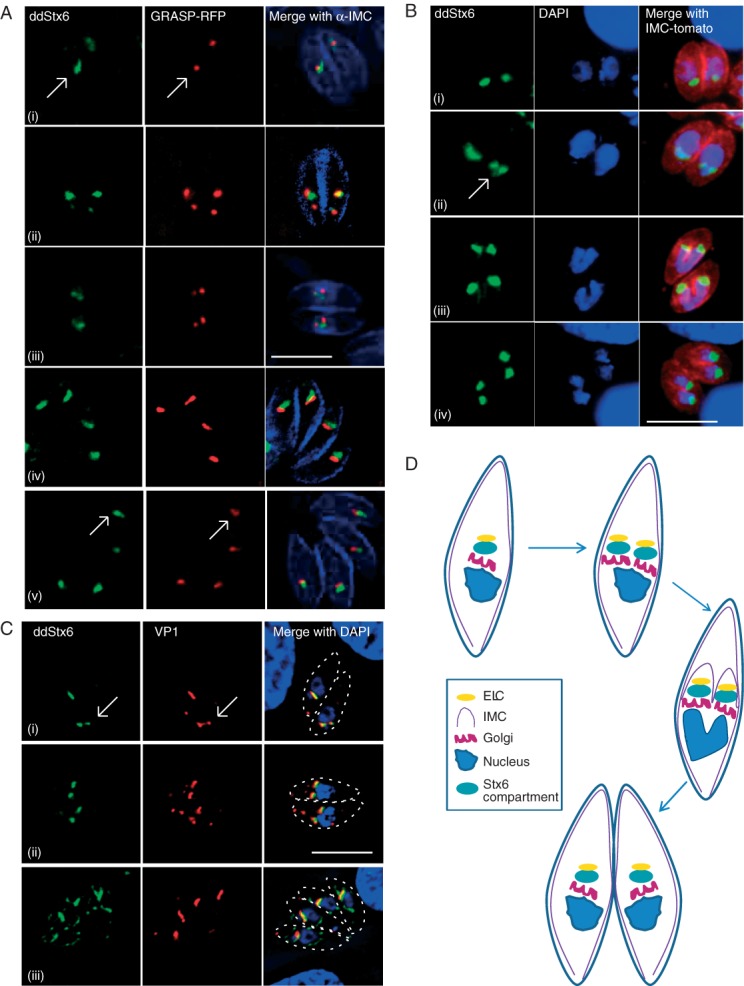
TgStx6 compartment replicates with the Golgi and ELC prior to nuclear division. A) ddStx6 parasites transiently transfected with GRASP-RFP to visualize the Golgi and stained with antibodies against the IMC (blue). (i and iv) Prior to replication, parasites have one Golgi and one TgStx6 compartment. (ii and v) The TgStx6 compartment replicates with the Golgi. White arrow indicates a parasite with two Golgi and two TgStx6 compartments. (iii) Newly forming daughter parasites contain two Golgi and two TgStx6 compartments. B) ddStx6 parasites stably transfected with IMC-tomato. Division of the TgStx6 compartment occurs prior to nuclear division. (i) Parasites with one nucleus and one TgStx6 compartment. (ii) TgStx6 compartment division. White arrow points to cell with one nucleus and two TgStx6 compartments. (iii) Nuclear division with two TgStx6 compartments. Daughter parasite IMC is clearly visible within the mother. (iv) After one division there are two daughter cells with two TgStx6 compartments and two nuclei. C) ddStx6 parasites stained with DAPI and antibodies against VP1, which indicate the ELC. (i) The TgStx6 compartment also replicates with the ELC. Replication is not synchronous across parasites within one PV. White arrow indicates a parasite with two ELC and two TgStx6 compartments. (ii) Prior to nuclear division, each parasite has two TGN and two ELC. (iii) After endodyogeny, each parasite contains one TgStx6 compartment and one ELC. Scale bars indicate 5 μM. D) Schematic of Golgi (magenta), TgStx6 compartment (green), IMC (purple), ELC (yellow) and nuclear (blue) replication.

Next, we analysed the behaviour of the TgStx6 compartment during the cell cycle of *T. gondii*. We performed colocalization studies using GRASP-RFP as a Golgi marker and antibodies against the IMC protein 1 (IMC1) (37) to investigate intracellular parasites at different stages during endodyogeny (Figure 2A). Although we never observed a difference in the number of Golgi and TgStx6 compartments, indicating a tight linkage of their segregation, it appeared that the TgStx6 compartment elongates prior to the Golgi at the early stages of endodyogeny (Figure 2A, arrows). This indicates that segregation of the TgStx6 compartment might be initiated prior to the Golgi. We also analysed the segregation of the TgStx6 compartment in relation to nuclear division and found that segregation occurs prior to nuclear division (Figure 2B, arrow). When we analysed the position of the TgStx6 compartment in relation to ELC during endodyogeny, we found a similar, tight linkage, although VP1 staining appeared to be more heterogeneous (Figure 2C). Together, these results indicate that segregation of the TgStx6 compartment and of the endomembrane system, including the Golgi and ELC, is tightly linked and occurs prior to nuclear division (Figure 2D), as described previously for the Golgi (6).

### TgStx6 localizes to the TGN

**Figure 3:**
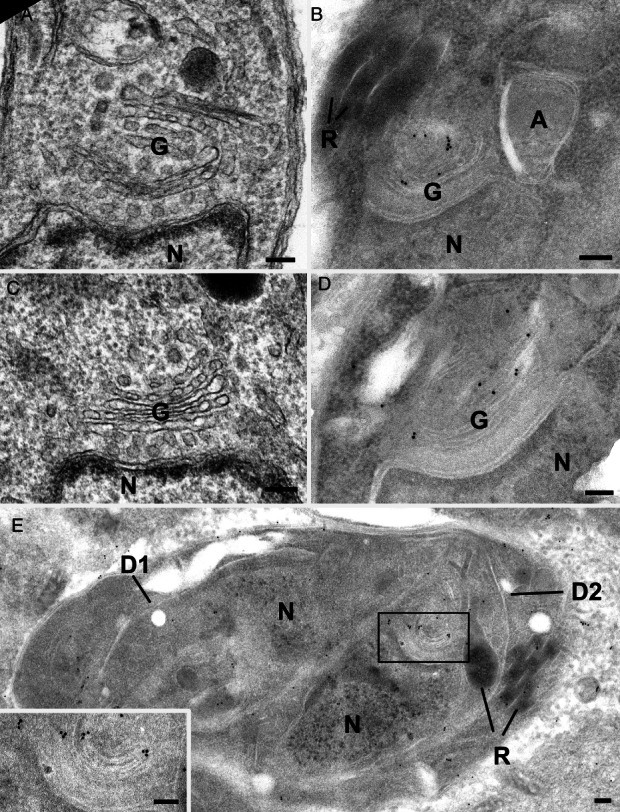
Evidence for the TGN. Routine EM (A, C) and immuno-EM (B–D) of ddStx6 parasites. Immunolabelled with anti-GFP and was visualized with 10-nm gold particles. Scale bars are 100 nm. A, C) Sections through the Golgi body (G) located just anterior to the nucleus (N) showing it to consist of a stack of flattened vacuoles. B, D) Sections through the perinuclear region (similar regions to those in A and C) showing the gold particles associated with the apical stack of the Golgi body. G, Golgi body; N, nucleus; A, apicoplast; R, rhoptry. D) Section through a parasite with two partially formed daughters (D1 and D2) showing gold particles associated with the apical side of a daughter Golgi body (enclosed area). N, nucleus. Inset: Enlargement of the enclosed area in D showing gold particles associated with apical stack of the Golgi body.

We speculated that TgStx6 localized to the elusive EEs in *T. gondii* or, alternatively, that apicomplexan parasites, similar to plants, lack EEs and instead have an elaborate TGN that directly receives endocytic vesicles from the plasma membrane and the Golgi (17). To pinpoint the position of the ddStx6 compartment we performed an ultrastructural analysis by immuno-electron microscopy (EM) using gold-labelled antibodies against GFP to detect ddStx6. In routinely processed samples the Golgi apparatus consists of a number of stacked membranes, with, in certain cases, the most apical membrane appearing slightly less concave (Figure 3A,C). In parasites processed for immuno-EM it was observed that gold particles were consistently and specifically located in the region of the outer most stack of the Golgi apparatus (Figure 3B,D). Similar labelling of the apical Golgi region was observed within the daughters formed by endodyogeny (Figure 3C).

In summary, we conclude that TgStx6 is likely to cycle between the TGN and ELC in *T. gondii*. Our ultrastructural analysis did not retrieve any conclusive evidence for the presence of EEs in these parasites, and we therefore favour the hypothesis that apicomplexan parasites contain a plant-like secretory system, with a TGN instead of EEs, and that the Stx6 compartment is likely the TGN. This TGN appears to be reduced, and is derived from and attached to the *trans*-most Golgi cisterna.

### TgStx6 is essential for parasite survival

Overexpression is regularly used to specifically inhibit the transport pathways of SNARE proteins (38–41). Indeed, overexpression of TgStx6 is not tolerated by the parasite (Figures S1 and 4A), indicating that this protein plays an essential role in the vesicular traffic of *T. gondii*. We performed a growth assay of ddStx6 parasites in the presence of different concentration of the inducer Shld-1 and found a complete block in parasite growth in the presence of 1 μM Shld-1 (Figure 4A). Stabilization of ddStx6 resulted in a strong signal apical to the nucleus and a weak signal at the surface of the parasite (Figure 4B). Immuno-EM also showed a strong perinuclear (Golgi region) localization of gold particles and also some labelling of the pellicular IMC (Figure 5C,D). This is consistent with the results obtained in transient assays (Figure S1A). Western blot analysis confirmed rapid regulation of ddStx6 in the presence of Shld-1. Significant upregulation was detected as early as 1 h postinduction and reached a maximum after 6 h (Figure 4C). The gross morphology of the parasites, when visualized with an antibody against aldolase, was altered upon addition of Shld-1. Well-distributed rosettes were not seen and the posterior ends of the parasites remained fused (Figure S1B).

### Stx6 overexpression does not affect invasion or egress but causes a block in daughter cell assembly and replication

To analyse the effect of ddStx6 overexpression on specific stages of the lytic cycle, we analysed invasion, replication and egress of parasites. To follow replication, parasites were grown for 24 h in the presence or absence of Shld-1 before the number of parasites per PV was counted (Figure 4D). We observed a large proportion of vacuoles with two or four parasites in the presence of Shld-1 (both 47.6%). In contrast, there were only a minor proportion of PVs (4.7%) in the 8-parasite stage and no PVs in the 16-parasite stage, whereas replication in the absence of Shld-1 appeared to be normal with 28, 42.3, 28.3 and 1.4% of PVs in the 2-, 4-, 8-and 16-parasite stage, respectively. Similar values were obtained for RHΔHX control parasites. This indicates that the observed growth defect of parasites overexpressing ddStx6 is owing to a delay in parasite replication. Importantly, longer incubation results in the formation of morphologically abnormal parasites (Figures S1 and S2), indicating that interference with TgStx6 function causes accumulative and pleiomorphic defects that ultimately lead to a complete block in productive replication.

The ddFKBP system allows a rapid regulation of protein levels and enables the investigation of protein functions in invasion and egress even after short induction times (35). As prolonged overexpression of ddStx6 leads to the formation of structurally abnormal parasites that are unable to invade when released from the host cell (data not shown), we performed invasion assays after 6-h treatment with Shld-1, when ddStx6 has been maximally induced (Figure 4E). We were unable to observe a significant difference in invasion, in either the presence or absence of Shld-1 (92 and 91%, respectively), and in comparison to wild-type RHΔHX parasites (−Shld-1, 95%; +Shld-1, 96%; Figure 4E). Similarly, induced host cell egress (42) was not blocked when parasites were treated with Shld-1 for 6 h (Figure 4F, iv). In stark contrast, when induced ddStx6 parasites were allowed to replicate once (induction for 12 h) they were unable to egress as the daughter parasites are not properly formed during endodyogeny (Figure 4F, v). Together with the IFA, these data indicate that TgStx6-dependent vesicular transport is not directly involved in host-cell invasion or egress.

### TgStx6 overexpression causes fragmentation of the Golgi stack

To visualize the effect of TgStx6 overexpression on the Golgi we analysed the Golgi of parasites grown in the presence and absence of Shld-1 for 16 h by EM. When we examined the Golgi region of 25 parasites in each group, the structure of the Golgi was significantly changed when the parasites were grown in Shld-1. In the control parasites the Golgi body consisted of a typical membranous stacks located anterior to the nucleus (Figure 3A,C). In contrast, the Golgi of the overexpressing TgStx6 parasites had a vesicular appearance with loss of many or all the membranous stacks (Figure 5B,C).

We then performed an immunofluorescence time course on the effect of ddStx6 overexpression on the Golgi, with GRASP-RFP as a marker for the morphology and segregation of this organelle. ddStx6 parasites were allowed to invade for 1 h and were incubated in the presence or absence of the inducer for 12, 18, 24 and 30 h prior to fixation. In contrast to the ultrastructural analysis, we did not observe a significant effect on the distribution of GRASP-RFP (Figure 5E). In all cases, we observed a single signal close to the nucleus, which indicates normal Golgi location. By immunofluorescence, the TgStx6 compartment itself expanded significantly and lost its sharp definition in a time-dependent manner. This result indicates a block of vesicular transport from the TGN. Interestingly, the fragmentation of the Golgi seen in EM seems to have no influence on anterograde transport of vesicles to the secretory organelles, because we were unable to detect significant effects on the morphology or biogenesis of the unique apicomplexan secretory organelles (micronemes and rhoptries, Figure S3). This situation appears to be very similar to observations made in other eukaryotes (43,44).

### TgStx6 overexpression causes expansion of the ELC

**Figure 6:**
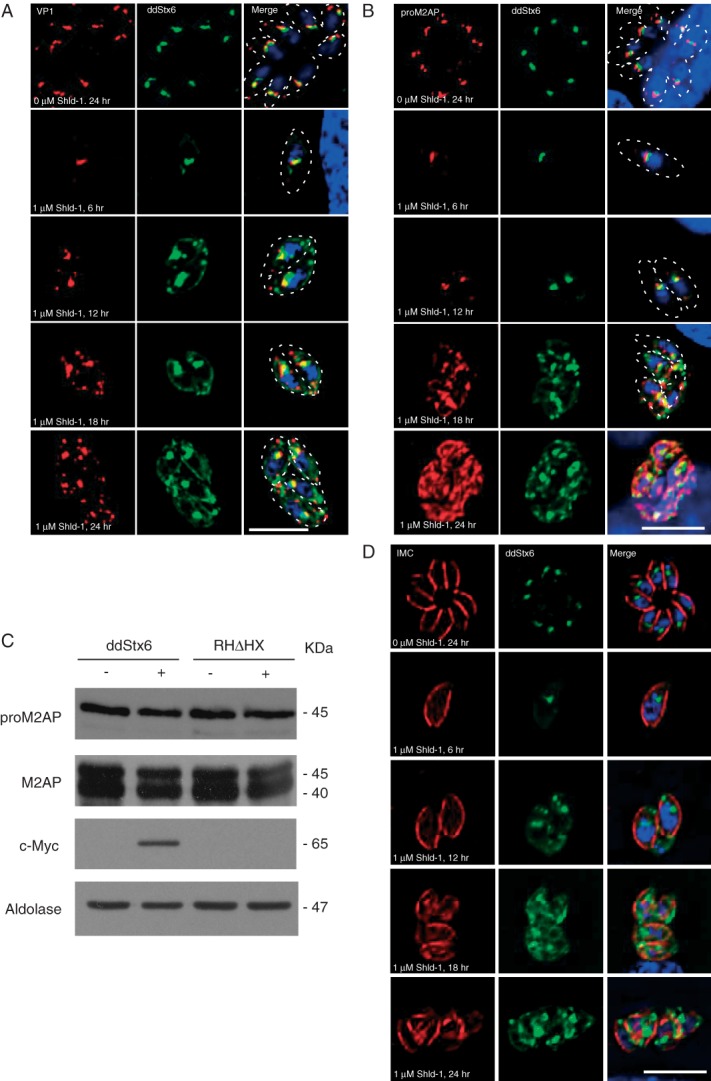
Overexpression of TgStx6 causes expansion of the ELC. A) ddStx6 parasites stained with α-VP1; at 18 h there is minor fragmentation of ELC, which becomes more pronounced by 24 h. B) ddStx6 parasites stained with α-proM2AP. At 18 h expansion of proM2AP signal can be detected; by 24 h postinduction any tertiary structure is lost. C) Processing of parasite proteins that traffic through the endosomes remains normal upon ddStx6 overexpression. Western blots of ddStx6 parasite lysates (−/+Shld-1) probed with antibodies against proM2AP, M2AP, c-Myc and aldolase (loading control). D) The IMC is defective at the posterior end upon the addition of Shld-1; parasites labelled with antibodies against IMC. Scale bars are 5 μM.

We then investigated the effects of TgStx6 overexpression on ELC using antibodies against VP1 and proM2AP as markers (11,13) in a time course. ddStx6 parasites were allowed to invade for 1 h and were incubated in the presence or absence of Shld-1 for 6, 12, 18 and 24 h prior to fixation. VP1 staining did not significantly alter upon Shld-1 induction for up to 12 h (Figure 6A). At 18 and 24 h postinduction, VP1 distribution was altered in the majority of parasites. It became expanded, and a tubulated VP1 signal was observed throughout the parasites, while still maintaining a concentrated postnuclear signal. We found a more striking effect when we examined the proM2AP staining (Figure 6B). At 6 h postinvasion, the proM2AP staining was not significantly affected and only slightly expanded. However, after 12 h there was a significant expansion in all parasites. By 18–24 h postinduction, the usually sharp defined structure was lost and there appeared to be a huge expansion and fragmentation of this compartment (Figure 6B).

Several secretory proteins are proteolytically matured in the ELC (7). Given the dramatic effect on ELC morphology upon ddStx6 expression, we wanted to test if protein processing was abolished. Western blot analysis of M2AP and proM2AP in RHΔHX and ddStx6 parasites showed that proM2AP is processed correctly to its mature form, M2AP, when ddStx6 is induced for 6 h (Figure 6C), indicating that proteolytic processing and maturation of micronemal proteins are not affected in ddStx6-overexpressing parasites. In good agreement with this result, we were unable to observe a block in the transport or distribution of micronemal or rhoptry proteins. Although TgStx6 overexpression caused parasites to become disorganized, owing to a defect in IMC maturation, micronemes and rhoptries were still formed (Figure S3). Together, these results indicate that TgStx6 is required for retrograde transport from ELC to the TGN, and from the TGN to the Golgi, which is also in line with our localization study. Furthermore, it appears that the expansion and fragmentation of ELC do not abrogate vesicular transport to the micronemes.

### TgStx6 overexpression causes a maturation defect of the IMC

The IMC of apicomplexan parasites is assembled in a stepwise process that involves two independent Rab-GTPases, Rab11A and Rab11B (45,46). IFA of the consequence of TgStx6 overexpression on the IMC over time showed an effect on IMC organization and development as early as 6 h postinduction (Figure 6D), where the posterior end of the IMC appeared disorganized.

**Figure 7:**
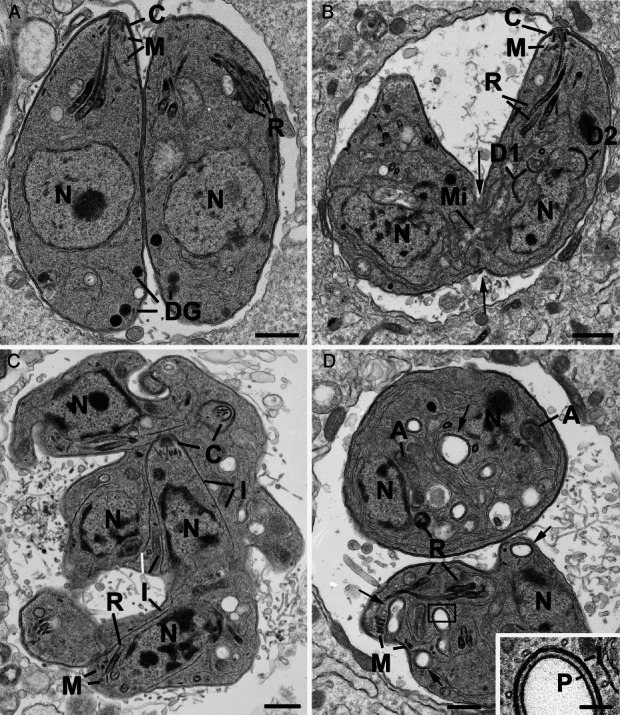
Parasite ultrastructure is perturbed upon ddStx6 overexpression. EM images of ddStx6 parasites (−/+Shld-1) 16 h postinvasion. Scale bars are 500 nM (A–D) and 100 nm (inset). A) ddStx6 parasite (−Shld-1) showing the daughters formed by endodyogeny exhibiting the apical organelles of the conoid (C), rhoptries (R) and micronemes (M). Note the dense granules (DG) in the basal cytoplasm. N, nucleus. B–D) ddStx6 parasites overexpressing TgStx6 (+1 μM Shld-1). B) Longitudinal section through two incompletely formed daughters that are still connected at the posterior (arrows) while the daughter IMC is enlarged (D1 and D2) as the next cycle of endodyogeny has been initiated. The conoid (C), rhoptries (R) and micronemes (M) could be identified but dense granules were not observed. Mi, mitochondria. C) Section through an irregularly shaped multinucleate (N) parasite showing apical organelles and partially formed and disorganized IMC (I) of multiple daughters. N, nucleus; C, conoid; M, micronemes; R, rhoptry. D) Section through two daughters showing abnormal cytoplasmic features with randomly distributed rhoptries (R) and micronemes (M) and areas of pellicle formation within the cytoplasm (arrows). A, apicoplast; N, nucleus. Inset: Details of the enclosed area in D showing the pellicular structure consisting of the outer plasma membrane (P) and the IMC (I) with associated microtubules.

As seen in our IFA, abnormalities in the IMC and pellicle formation were also observed during ultrastructural analysis. These changes appeared to relate to the stage of IMC formation at the time of TgStx6 overexpression. For example, if overexpression occurred late in endodyogeny when IMC formation was already concluded, it appeared to prevent completion of the pellicle formation in a similar manner to parasites expressing dominant negative Rab11A in terms of IMC maturation (Figure 7B) (46). TgStx6 overexpression at earlier stages of IMC formation resulted in a disorganized IMC orientation more similar to that seen in MORN1 knockout parasites (47,48) (Figure 7C). At 24 h postinduction, where further rounds of endodyogeny had proceeded, there was evidence of pellicle formation within the daughter cytoplasm consisting of plasma membrane, IMC and subpellicular microtubules (Figure 7D, inset). This inability of daughter parasites to separate, as observed in the case of Rab11A (46), causes a certain loss of polarity and therefore a minor pleiomorphic effect on micronemes and rhoptry staining (see Figure S3A,D, 24 h). However, in good agreement with our immunofluorescence time courses, where we looked at the effect of TgStx6 overexpression on Rop5 and M2AP localization (Figure S3A,D), our ultrastructural analysis did not highlight any significant differences in the micronemes or rhoptry organelles, indicating that TgStx6 is not directly involved in vesicular transport to these apical organelles.

We also analysed apicoplast formation, as transport to the apicoplast was previously shown to be linked to the ELC (49). Immunostaining parasites with the apicoplast antibody HSP60 (Figure S3B) revealed no difference in the apicoplast size or location when TgStx6 was induced. Analysis of the fluorescently tagged apicoplast marker, FNR-RFP, which was transfected into the ddStx6 parasites, also showed no alterations in the presence of Shld-1 (data not shown). This was similar to that observed by EM (Figure 7D).

Dense granular proteins have been implicated in trafficking from the Golgi and ELC to the PV (50). Therefore, we performed IFA to determine if ddStx6 overexpression affected dense granule protein secretion. We stained parasites grown in the absence and presence of Shld-1 for 18 h with antibodies against the dense granule protein Gra9. Irrespective of the level of ddStx6 expression, Gra9 was secreted normally into the PV (Figure S3C), which indicates that trafficking from the dense granules to the parasite surface does not involve TgStx6. In contrast, when examined by EM, dense granules were readily identified in untreated parasites (Figure 7A) but there appeared to be a marked reduction in the formation of dense granules in the Shld-treated parasites (Figure 7B–D). To quantify the difference, a random sample of 30 parasites from the +Shld and −Shld samples were evaluated for the presence of one or more dense granules. It was observed that, while over 50% of untreated parasites had dense granules, less than 10% of parasites overexpressing TgStx6 had dense granules. Therefore, we speculate that TgStx6 is involved in trafficking from the TGN to the dense granules, and a block in this pathway interrupts trafficking to these organelles.

## Discussion

Vesicular transport plays an essential role in all eukaryotes for the biogenesis, maintenance, communication of intracellular compartments and motility (51,52). Most of our knowledge on the organization of the vesicular trafficking system is based on studies performed in yeast and, to a lesser extent, plants. Although it is tempting to use findings from these model systems as a blueprint for other eukaryotes, one should keep in mind that the organization of the endomembrane system of diverse eukaryotic taxa is not conserved, and shows multiple secondary losses and gene duplications (53,54). Not only can organelles be unique to certain taxa, but also the molecular machineries involved in specific trafficking steps might differ. For example, it has been convincingly demonstrated that the mechanisms involved in clathrin-mediated endocytosis and the involvement of dynamin in this step are an example of convergent evolution between ciliates and animals (55). Similarly, it has been demonstrated in the kinetoplastida *Trypanosoma brucei* that clathrin, but not dynamin, is required for endocytosis (56,57). Furthermore, a recent study in *T. gondii* demonstrated that a homologue of VPS10, a sortilin-like receptor, is essential to the parasite, unlike its counterparts in other systems (10). Therefore, a systematic, functional comparison of conserved factors in different lineages will contribute to complete our picture of the endomembrane system organization within different lineages.

One example of lineage-specific differences is the involvement of endosomal compartments in endocytic and secretory traffic, as seen, for example, in the case of animals, plants and kinetoplastida (18,57). In the case of apicomplexan parasites, endosomes are meagrely characterized. Recent studies demonstrate the presence of a vacuolar-like compartment in *T. gondii* that is linked to the ELC, based on colocalization studies with Rab7 (11,13). While immunolocalization analysis convincingly demonstrates the presence of this prominent vacuole, the nature of EEs remains inadequately defined and their existence rather indirectly demonstrated. Evidence includes the observation that overexpression of some micronemal proteins results in accumulation in a post-Golgi compartment (58), the location of Rab5A at this compartment (9) and the identification of processing events in this organelle (7).

To define the secretory and endocytic system of apicomplexan parasites in more detail, we performed a bioinformatic search for highly conserved factors in plants, animals, kinetoplastida and alveolates, focussing on Stx6, because it is well described as a sorting factor at the intersection of the TGN and EEs (23,24). Here, we demonstrate that TgStx6 is a highly conserved SNARE in apicomplexan parasites with a well-conserved Habc and TM domain. We show that TgStx6 localizes anterior to a classical Golgi structure, indicating the presence of an elaborate TGN in apicomplexan parasites, similar to plants. In contrast, we were unable to identify structures that could correspond to classical endosomes in immuno-EM analysis, and we speculate that the TGN of *T. gondii* acts as an EE, where secretory and possibly endocytic traffic meets. Duplication of the TGN appears to be tightly linked to the Golgi during parasite replication. Unfortunately, our efforts to perform a detailed time-lapse analysis of parasite replication failed owing to technical problems resulting from photobleaching of the GRASP-RFP signal.

Our functional analysis of TgStx6 demonstrates that this protein most likely plays an important role in retrograde protein transport from the ELC to the TGN and Golgi. Overexpression results in Golgi fragmentation and a significant expansion of the ELC. This is similar to the role observed for Stx6 in other eukaryotes (25). In contrast, protein transport to the regulated secretory organelles (micronemes and rhoptries) was not significantly affected. Furthermore, overexpression of ddStx6 led to a defect in cytokinesis, with an aberrant block in IMC maturation. However, in contrast to specific IMC deformation, seen in the case of Rab11A or Rab11B mutants (45,46), overexpression of ddStx6 appears to have multiple effects on IMC development, possibly indicating a pleiotropic effect on Golgi and ELC function during IMC development. Although the observed peripheral location of TgStx6 might suggest a direct role in vesicle delivery to the IMC, our current data do not allow a firm conclusion at this point. Prolonged overexpression of ddStx6 causes pleiomorphic effects, probably owing to a complete breakdown of the parasites' secretory system, affecting all parasite organelles and ultimately leading to parasite death. Finally, overexpression of TgStx6 caused a block in trafficking from the TGN to the dense granules. Future experiments and a detailed characterization of the remaining apicomplexan SNAREs will allow us to pinpoint the pathways involved in protein transport to the individual organelles.

Meanwhile, it appears that the early secretory system of *T. gondii* has a plant-like configuration, where a TGN also fulfils early endosomal functions with TgStx6 as an essential, highly conserved part of the involved transport steps. We suggest TgStx6 as a TGN marker, and that the TGN is distinct from the ELC. Therefore, it appears that this branch of the vesicular trafficking system shows high functional conservation between eukaryotes from apicomplexan parasites, via plants, to humans.

## Materials and Methods

### Generation of constructs

Primers used in this study are listed in Table S2. Restriction sites used for cloning are indicated.

The *HA-Stx6* and *GFP-Stx6* constructs were a kind gift from Giel van Doreen. To generate the ddStx6 construct *ddFKBP-myc-GFP-TgStx6-CAT*, the destabilization domain, myc tag and some plasmid backbone were removed from the *pTub-destab106YFP-CAT* plasmid (35) and placed downstream of the *GFP-Stx6* fusion in the *GFP-Stx6* construct using the AvrII and PacI restriction sites.

The vector *loxPStx6loxPYFP-HX* was generated by modifying the parental Gene-swap vector *p5RT70loxPKillerRedloxPYFP-HX* in three steps (34). First, the *stx6* 3′UTR was amplified from genomic DNA using the primer pair 3′UTRStx6fw/rv. The PCR fragment was cloned into *p5RT70loxPKillerRedloxPYFP-HX* via SacI. Second, the N-terminally HA-tagged *stx6* ORF (TGME49_300240) was generated by Eurofins MWG Operon (Germany) using the *HA-Stx6* vector sequence as a template. *HA-stx6* was cloned using EcoRI and Pac1. Finally, the *stx6* 5′UTR containing the endogenous promoter was amplified from genomic DNA using the primer pair 5′UTRStx6fw/rv and cloned into the final vector using EcoRI and AvrII.

### Multiple sequence alignment

Protein sequences were retrieved from NCBI/GenBank, http://ToxoDB.org, http://PlasmoDB.org and http://www.OrthoMCL.org (59). After randomizing sequence order, alignments were created with ClustalX v1.83 using the default Pairwise and Multiple Alignment Parameters. *Saccharomyces cerevisiae* was used as the source organism to study TgStx6 interactors. The yeast Tlg1p interactors were downloaded from http://www.pathwaycommons.org. Interactions represented as networks using Cytoscape (http://www.cytoscape.org/).

### *Toxoplasma gondii* cultivation and transfection

*Toxoplasma gondii* parasites (RH*hxgprt*^–^, RHΔHX or *ku80::hx::diCre* parasites) were grown in human foreskin fibroblast (HFF) cells and maintained in Dulbecco's modified Eagle's medium (DMEM, PAA) supplemented with 10% foetal calf serum, 2 mM glutamine and 25 mg/mL gentamycin. For the generation of stable transformants, 5 × 10^7^ freshly released parasites were transfected by electroporation with 60 µg of linearized plasmid DNA and selected in the presence of chloramphenicol (1 μM in EtOH; *CAT*), pyrimethamine (1 μM in EtOH; *PYR*) or mycophenolic acid (12.5 mg/mL in MeOH; MPA)/xanthine (20 mg/mL in 1 M KOH; XAN), as described previously (60,61). To study the localization and replication of TgStx6 in relation to the Golgi, ddStx6 and Stx6-YFP parasites were transiently transfected with 60 µg of the *pTubGRASP55-RFP*/*sagCAT* (GRASP-RFP) plasmid (62). To study nuclear replication, ddStx6 parasites were cotransfected with 40 µg of linearized IMC-tomato plasmid (a kind gift from Boris Striepen) and 20 µg of a linearized plasmid containing dihydrofolate reductase (61), and selected using 1 mM pyrimethamine.

The parasite strain expressing *ha-stx6* under the control of its endogenous promoter (*ku80::diCre*/*pstx6-hastx6*, referred to here as pstx6-HAStx6) was generated by transfecting 50 µg of the plasmid *loxPStx6loxPYFP-HX* into the *ku80::diCre* parasites. After transfection parasites were selected for stable integration using XAN and MPA, as described previously (60). After the selection process the parasite pool was serially diluted to isolate single clones.

### Immunofluorescence analysis

For IFA, HFF cells grown on coverslips were inoculated with scratched *T. gondii* parasites in the absence or presence of 1 μM Shld-1 for 1 h before washing and left to grow for 6, 12, 18 and 24 h. Cells were fixed either with 4% w/v paraformaldehyde in phosphate-buffered saline (PBS) for 20 min at room temperature or 100% ice-cold methanol for 10 min at −20°C. Cells fixed with paraformaldehyde were permeabilized and blocked with 0.2% Triton X-100 and 2% w/v bovine serum albumin (BSA) in PBS for 20 min. The staining was performed using various primary antibodies against different organelles: (IMC 1:1500; M2AP 1:1000; aldolase 1:2000; proM2AP 1:500; SAG1 T91E5 1:1000; ROP5 T53E2 1:1000; HA 1:100; GRA9 1:500; apicoplast HSP60 1:1000; GFP 1:500) for 60 min followed by secondary Alexa Fluor 488, Alexa Fluor 594 or Alexa Fluor 350 conjugated antibodies for another 45 min (1:3000, Life Technologies–Molecular Probes).

For image acquisition z-stacks of 2-µm increments were collected using a UPLSAPO 100× oil (1.40 NA) objective on a Deltavision Core microscope (Image Solutions—Applied Precision, GE) attached to a CoolSNAP HQ^2^ CCD camera. Deconvolution was performed using SoftWoRx Suite 2.0 (Applied Precision, GE) and further processed using ImageJ 1.34r software and Photoshop (Adobe Systems Inc.). Image acquisition was also conducted using a 100× oil objective on a Zeiss Axioskope 2 MOT+ microscope attached to a Hamamatsu Orca-ER digital CCD camera using OpenLab 5.5.2 software (Improvision) and further processed using ImageJ and Adobe Photoshop.

### Immunoblot analysis

For immunoblot analysis intracellular parasites were cultivated in HFF cells in the absence or presence of 0.1–1 mM Shld-1 for up to 6 h. Subsequently, parasites were harvested and washed in ice-cold PBS, and the pellet was resuspended in 4× LDS (lithium dodecyl sulphate) loading buffer containing 1× reducing agent (Invitrogen) in PBS. The volume was adjusted so that approximately 1 × 10^6^ parasites were loaded per lane on a 12% SDS–PAGE using reducing conditions with 100 mM DTT. The lysate was boiled before loading. SDS–PAGE and western blot analysis were performed as described previously (40). For detection, polyclonal anti-myc (1:500, Affinity BioReagents), anti-M2AP (1:2000) and anti-proM2AP (1:1000), anti-GFP (1:1000) antibodies were used. An anti-aldolase [1:10 000 (46)] antibody was used as an internal control.

### Growth assay

The growth assay was performed as previously described (63). Monolayers of HFF cells, grown in six-well plates, were infected with 1000 parasites per well in the absence or presence of 0.1–1 μM Shld-1. After 5 days of incubation at normal growth conditions (37°C, 5% CO_2_), cells were fixed for 10 min with 100% methanol, stained for 10 min with Giemsa stain and washed with PBS. Images were captured with a 4× objective on a Zeiss Axiovert 40 CFL microscope equipped with a Zeiss Axiocam ICC1 CCD camera using Axiovision LE 4.8.1 software. Images were further processed with Photoshop.

### Invasion assay

For the analysis of the parasites' ability to invade HFF monolayers, intracellular ddStx6 and RHΔHX parasites were preincubated in the absence and presence of 1 μM Shld-1 for 6 h. The cells were then scratched and passed through a 20-gauge needle before adjusting the parasites to 1 × 10^7^ per millilitre, and allowing them to invade fresh HFF monolayers grown on coverslips in the absence and presence of 1 μM Shld-1 for 1 h. Monolayers were then washed four times with PBS to remove any extracellular, unattached parasites, before fixation with 4% paraformaldehyde. To determine the invasion efficiency, IFA was performed using anti-SAG1 antibody (1:1000) prior to permeabilization to stain extracellular parasites, and then an anti-IMC antibody (1:1500) postpermeabilization to stain all parasites. The number of extracellular and intracellular parasites was counted and calculated as a percentage value of 100%.

### Replication assay

For the analysis of the parasites' replication ability, 4 × 10^5^ per millilitre of freshly released extracellular ddStx6 or RHΔHX parasites were preincubated in the absence and presence of 1 μM Shld-1 for 6 h. Subsequently, parasites were allowed to invade fresh HFF cells grown on coverslips and were incubated in the absence and presence of 1 μM Shld-1 for 24 h. To determine the replication efficiency, IFA was performed using the anti-aldolase antibody (1:2000), and the number of nuclei per parasite vacuole was counted and calculated as a percentage value of 100%.

### Egress assay

Freshly lysed parasites were allowed to invade HFF monolayers grown on coverslips for 36 h. Shld-1 was added at 6 and 12 h prior to artificially inducing egress with prewarmed egress media [DMEM supplemented with 2 mM glutamine, 25 mg/mL gentamycin and 1 μM A23187 (Sigma) calcium ionophore] for 10 min at 37°C in the dark. Parasites were fixed with 100% ice-cold methanol for 10 min and were stained with anti-SAG1 antibody (1:1000) after blocking with 2% BSA in PBS.

### Electron microscopy

Monolayers of HFF, grown on 6-cm dishes, were infected with ddStx6 parasites and cultured for 16 and 24 h in the absence or presence of 1 μM Shld-1 and subsequently fixed with 2.5% glutaraldehyde in 0.1 M phosphate buffer pH 7.4 (1 M Na_2_HPO_4_ and 1 M NaH_2_PO_4_). Samples were processed for routine EM as described previously (64). In summary, samples were postfixed in osmium tetroxide, dehydrated, treated with propylene oxide and embedded in Spurr's epoxy resin. Thin sections were stained with uranyl acetate and lead citrate prior to examination in a JEOL 1200EX electron microscope.

Samples for immuno-EM were fixed with 2% paraformaldehyde in 0.1 M phosphate buffer pH 7.4 and processed as described previously (65). In summary, the samples were dehydrated and embedded in LR White resin. Then sections were floated on drops of 1% BSA in PBS to block non-specific staining. Rabbit anti-GFP was washed and exposed to goat anti-rabbit Ig conjugated to 10-nm gold particles. Sections were stained with uranyl acetate prior to examination in the EM.

## Supporting Information

Figure S1 **Overexpression of TgStx6 is not tolerated by the parasite.** A) Schematic of the two full-length wild-type TgStx6 fusion proteins fused to either GFP or HA (HA-Stx6 and GFP-Stx6). Immunofluorescence images of parasites transiently transfected with HA-Stx6 and GFP-Stx6; HA-Stx6 was detected using an HA antibody. Both fusion proteins displayed a postnuclear localization, with some plasma membrane staining. We were unable to generate stable parasites using either construct. B) The gross morphology of the parasites overexpressing ddStx6 is altered. Epifluorescence and brightfield analysis of ddStx6 parasites (–/+Shld-1) stained with antibodies against aldolase. This figure is associated with Figures 1 and 4 and shows the initial constructs used in the study and their localization. It also shows the gross morphological effects of overexpressing TgStx6 on the parasite.

Figure S2 **Electron microscopy images of ddStx6 parasites (–/+Shld-1) 16 h postinvasion.** Most vacuoles containing untreated parasites have four parasites; Shld-1-treated PVs contain mainly two parasites. This figure is associated with Figure 4 and shows ultrastructural effects of overexpressing TgStx6 on the parasite.

Figure S3 **TgStx6 overexpression does not have a major effect on the apical organelles or the apicoplast.** Immunofluorescence analysis of ddStx6 parasites treated with (–/+)Shld-1 and fixed at 6, 12, 18 and 24 h postinvasion. A) Micronemes appear normal: parasites labelled with antibodies against M2AP. B) The apicoplast is not affected by ddStx6 overexpression: ddStx6 parasites stained with the apicoplast antibody HSP60. C) Dense granule secretion is not affected by ddStx6 overexpression at 18 h postinvasion; parasites stained with antibodies against GRA9. Scale bars are 5μM. D) Rhoptries appear slightly disturbed at 24 h: parasites probed with antibodies against Rop5. This figure is associated with Figures 5 and 7 and shows using immunofluorescence analysis the effect of overexpressing TgStx6 on the micronemes, apicoplast, dense granules and rhoptry organelles over time.

Table S1 Putative interaction factors of TgStx6. This table is associated with Figure 1 and shows a list of the putative interaction factors of TgStx6, their ToxoDB and OrthoMCL references and their function.

Table S2 Primers used in this study. Restriction sites used for cloning are indicated. This table is associated with the *Materials and Methods* section and shows the primers used to construct the *loxPStx6loxPYFP-HX* vector.
