# Supplementary figures and images for: Toxoplasma gondii Syntaxin 6 Is Required for Vesicular Transport Between Endosomal-Like Compartments and the Golgi Complex
Source: Traffic. 2013 Sep 12;14(11):1166–81. doi: 10.1111/tra.12102 (PMC3963449; doi:10.1111/tra.12102)

Jackson et. al. Supplementary Figure 1

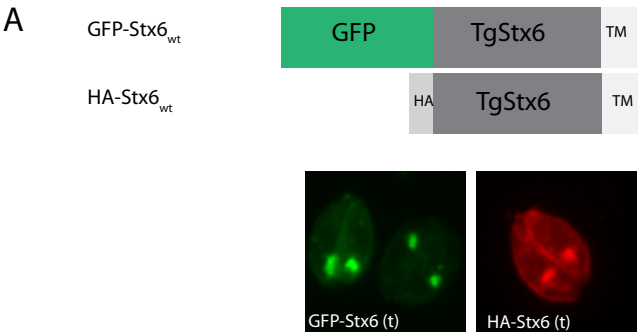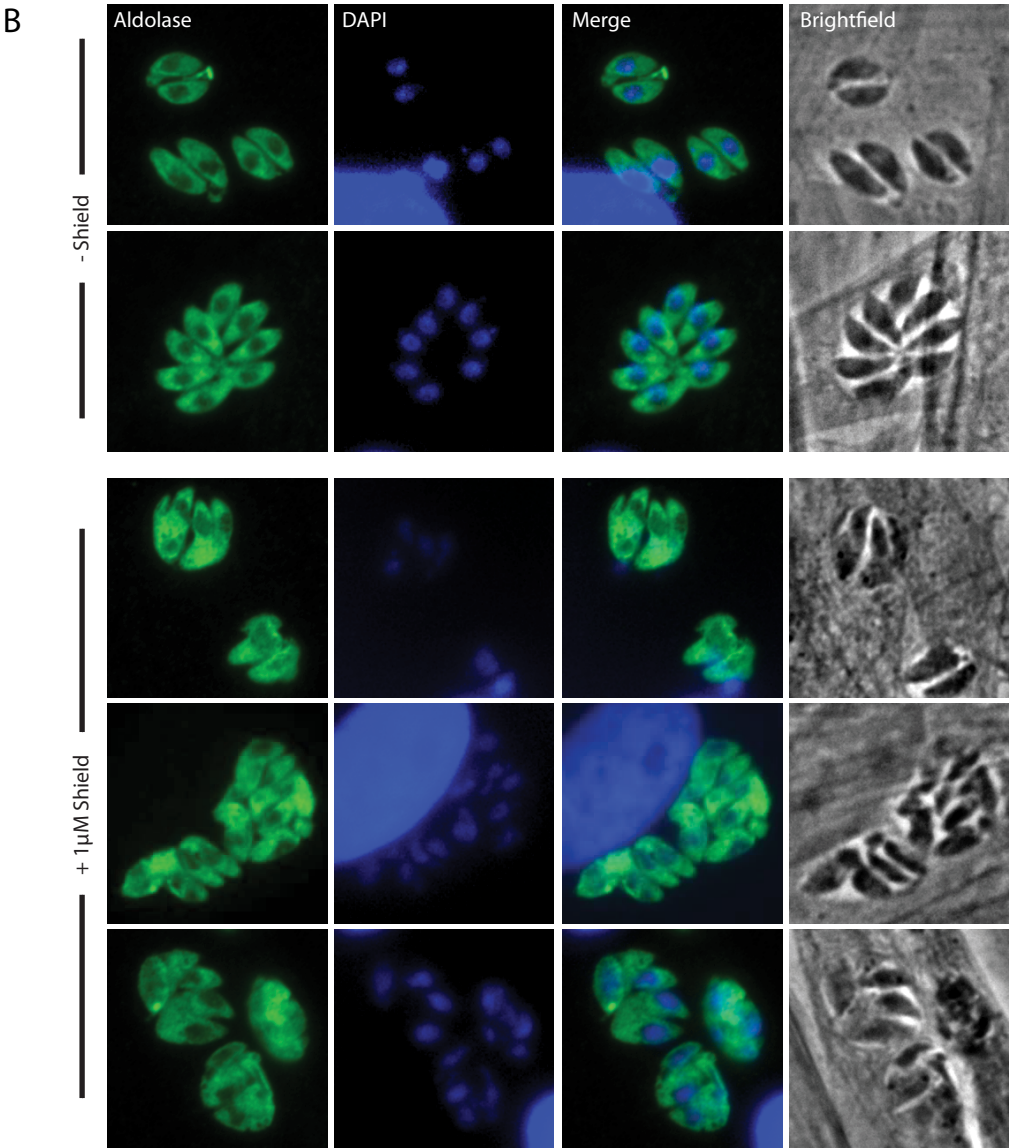

Supplement: Figure S1 — Overexpression of TgStx6 is not tolerated by the parasite. A) Schematic of the two full-length wild-type TgStx6 fusion proteins fused to either GFP or HA (HA-Stx6 and GFP-Stx6). Immunofluorescence images of parasites transiently transfected with HA-Stx6 and GFP-Stx6; HA-Stx6 was detected using an HA antibody. Both fusion proteins displayed a postnuclear localization, with some plasma membrane staining. We were unable to generate stable parasites using either construct. B) The gross morphology of the parasites overexpressing ddStx6 is altered. Epifluorescence and brightfield analysis of ddStx6 parasites (–/+Shld-1) stained with antibodies against aldolase. This figure is associated with Figures 1 and 4 and shows the initial constructs used in the study and their localization. It also shows the gross morphological effects of overexpressing TgStx6 on the parasite. [file tra0014-1166-sd1.pdf]

Jackson et. al. Supplementary Figure 2

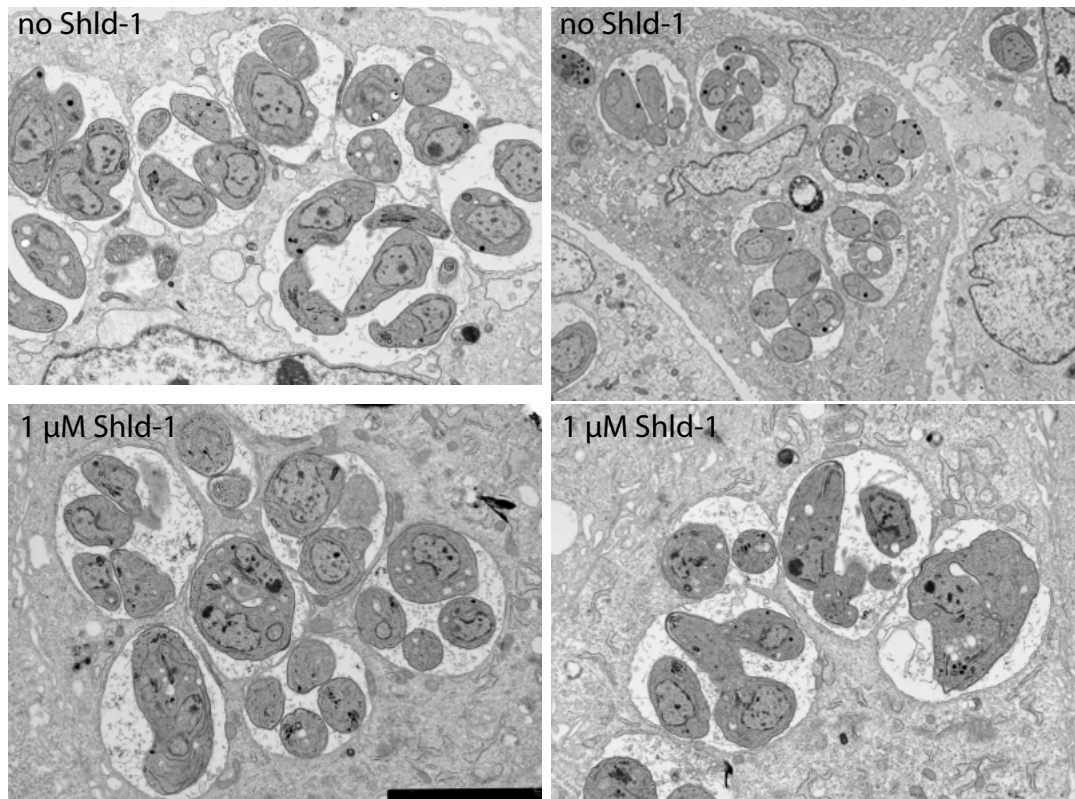

Supplement: Figure S2 — Electron microscopy images of ddStx6 parasites (–/+Shld-1) 16 h postinvasion. Most vacuoles containing untreated parasites have four parasites; Shld-1-treated PVs contain mainly two parasites. This figure is associated with Figure 4 and shows ultrastructural effects of overexpressing TgStx6 on the parasite. [file tra0014-1166-sd2.pdf]

A

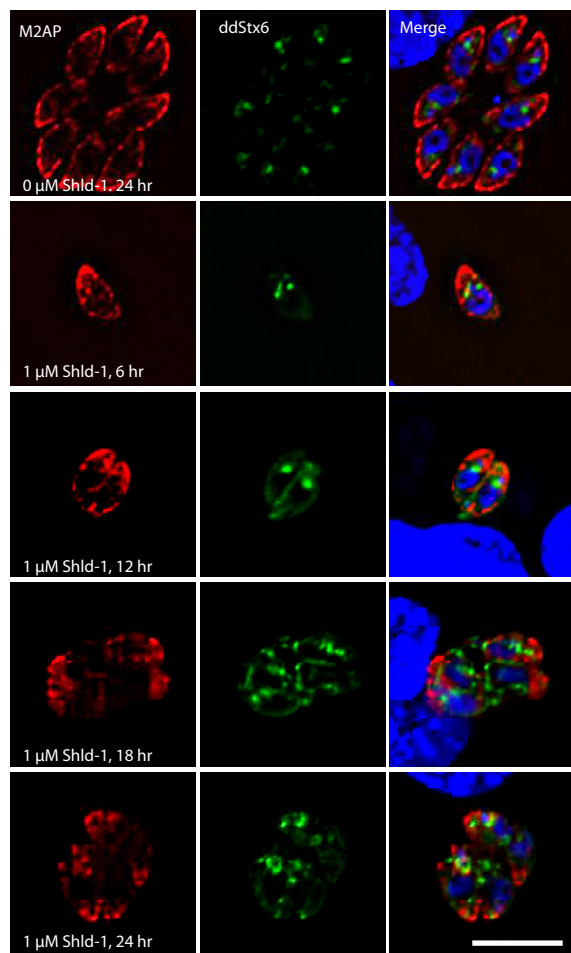

B

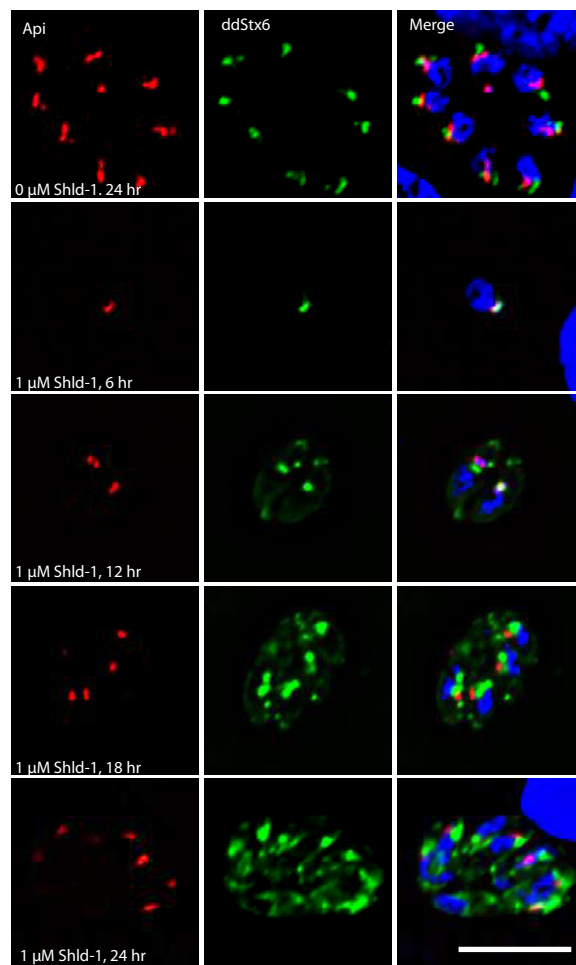

C

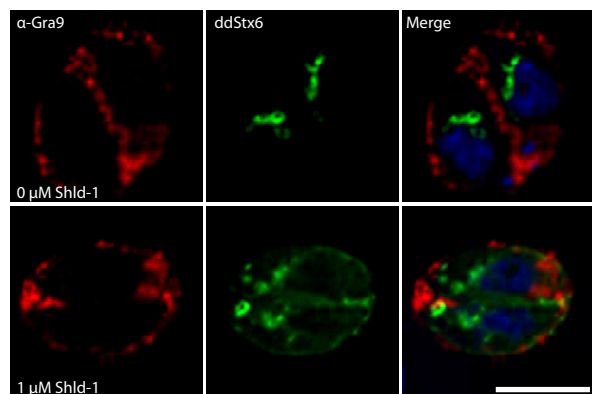

D

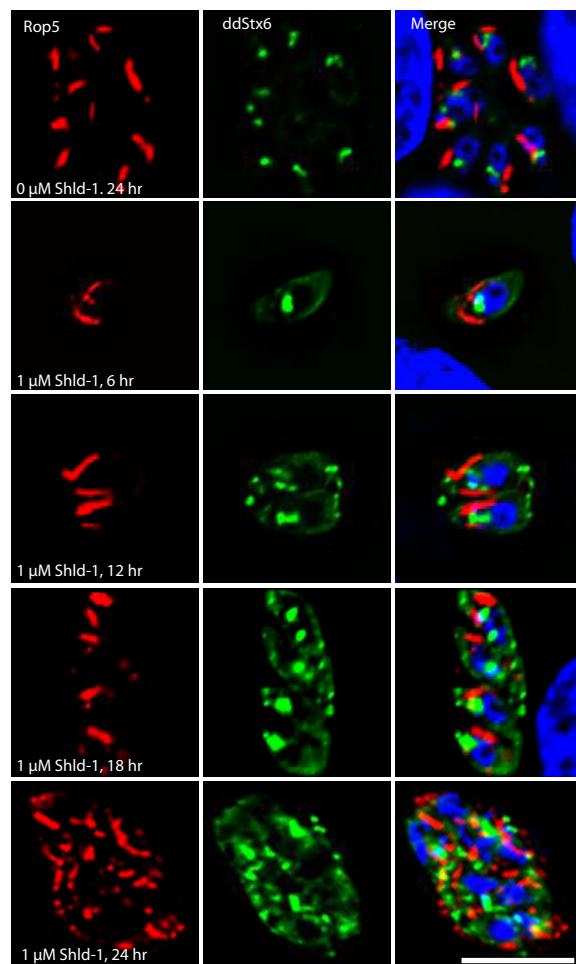

Supplement: Figure S3 — TgStx6 overexpression does not have a major effect on the apical organelles or the apicoplast. Immunofluorescence analysis of ddStx6 parasites treated with (–/+)Shld-1 and fixed at 6, 12, 18 and 24 h postinvasion. A) Micronemes appear normal: parasites labelled with antibodies against M2AP. B) The apicoplast is not affected by ddStx6 overexpression: ddStx6 parasites stained with the apicoplast antibody HSP60. C) Dense granule secretion is not affected by ddStx6 overexpression at 18 h postinvasion; parasites stained with antibodies against GRA9. Scale bars are 5μM. D) Rhoptries appear slightly disturbed at 24 h: parasites probed with antibodies against Rop5. This figure is associated with Figures 5 and 7 and shows using immunofluorescence analysis the effect of overexpressing TgStx6 on the micronemes, apicoplast, dense granules and rhoptry organelles over time. [file tra0014-1166-sd3.pdf]
