# Supplementary material for: Toxoplasma gondii Syntaxin 6 Is Required for Vesicular Transport Between Endosomal-Like Compartments and the Golgi Complex
Source: Traffic. 2013 Sep 12;14(11):1166–81. doi: 10.1111/tra.12102 (PMC3963449; doi:10.1111/tra.12102)
Supplement: Table S1 — Putative interaction factors of TgStx6. This table is associated with Figure 1 and shows a list of the putative interaction factors of TgStx6, their ToxoDB and OrthoMCL references and their function. [file tra0014-1166-sd4.pdf]

Table S1. Putative interaction factors of TgStx6.

Systematic Name Interactor A	Systematic Name Interactor B	Official Symbol Interactor A	Official Symbol Interactor B	ToxoDB	P-value	OrthoMLC	remark
YIL140W	YDR468C	AXL2	TLG1	-	-	OG5_137401	fungi
YGR142W	YDR468C	BTN2	TLG1	-	-	OG5_180477	fungi
YLR330W	YDR468C	CHS5	TLG1	-	-	OG5_136159	fungi
YNL051W	YDR468C	COG5	TLG1	-	-	OG5_139364	fungi
YNL041C	YDR468C	COG6	TLG1	-	-	OG5_129639	conserved, Golgi comlpex SU6
YER143W	YDR468C	DDI1	TLG1	TGME49_104680	1.90E-41	OG5_128104	DNA damage-inducible v-SNARE binding protein, contains a ubiquitin-associated (UBA) domain, may act as a negative regulator of constitutive exocytosis, may play a role in S-phase checkpoint control
YGL122C	YDR468C	NAB2	TLG1	-	-	OG5_136031	fungi
YLR039C	YDR468C	RIC1	TLG1	-	-	OG5_180333	fungi
YDL047W	YDR468C	SIT4	TLG1	TGME49_101010	4.00E-103	OG5_129276	S/T-phosphatase 2A
YDR515W	YDR468C	SLF1	TLG1	-	-	OG5_160982	RNA binding protein that associates with polysomes; proposed to be involved in regulating mRNA translation; involved in the copper-dependent mineralization of copper sulfide complexes on cell surface in cells cultured in copper salts in Plasmodium
YDR468C	YOL018C	TLG1	TLG2	TGME49_047930	3.00E-17	OG5_127496	Syntaxin-like t-SNARE that forms a complex with Tlg1p and Vti1p and mediates fusion of endosome-derived vesicles with the late Golgi; binds Vps45p, which prevents Tlg2p degradation and also facilitates t-SNARE complex formation
YDR468C	YMR197C	TLG1	VTI1	TGME49_042080	1.20E-135	OG5_127758	Vesicle transport v-SNARE protein;
YDR468C	YKR020W	TLG1	VPS51	-	-	OG5_155773	Component of the GARP (Golgi-associated retrograde protein) complex, Vps51p-Vps52p-Vps53p-Vps54p, which is required for the recycling of proteins from endosomes to the late Golgi; involved in localization of actin and chitin
YDR468C	YDR484W	TLG1	VPS52	TGME49_058830	2.80E-05	OG5_127921	Component of the GARP (Golgi-associated retrograde protein) complex, Vps51p-Vps52p-Vps53p-Vps54p, which is required for the recycling of proteins from endosomes to the late Golgi; involved in localization of actin and chitin
YDR468C	YOR036W	TLG1	PEP12	-	-	OG5_128082	Target membrane receptor (t-SNARE) for vesicular intermediates traveling between the Golgi apparatus and the vacuole; controls entry of biosynthetic, endocytic, and retrograde traffic into the prevacuolar compartment; syntaxin
YDR468C	YBL050W	TLG1	SEC17	TGME49_018760	5.10E-23	OG5_127387	Peripheral membrane protein required for vesicular transport between ER and Golgi and for the priming step in homotypic vacuole fusion, part of the cis-SNARE complex; has similarity to alpha-SNAP
YDR468C	YJL029C	TLG1	VPS53	TGME49_097230	5.70E-08	OG5_128060	Component of the GARP (Golgi-associated retrograde protein) complex, Vps51p-Vps52p-Vps53p-Vps54p, which is required for the recycling of proteins from endosomes to the late Golgi; required for vacuolar protein sorting

YDR468C	YDR027C	TLG1	VPS54	-	-	OG5_129527	Component of the GARP (Golgi-associated retrograde protein) complex, Vps51p-Vps52p-Vps53p-Vps54p, which is required for the recycling of proteins from endosomes to the late Golgi; potentially phosphorylated by Cdc28p
YDR468C	YLR026C	TLG1	SED5	TGME49_026600	8.10E-21	OG5_127926	cis-Golgi t-SNARE syntaxin required for vesicular transport between the ER and the Golgi complex, binds at least 9 SNARE proteins
YDR468C	YAL030W	TLG1	SNC1	TGME49_048100	2.40E-09	OG5_126859	Vesicle membrane receptor protein (v-SNARE) involved in the fusion between Golgi-derived secretory vesicles with the plasma membrane; member of the synaptobrevin/VAMP family of R-type v-SNARE proteins
YDR468C	YOR327C	TLG1	SNC2	-	-	OG5_126859	Vesicle membrane receptor protein (v-SNARE) involved in the fusion between Golgi-derived secretory vesicles with the plasma membrane; member of the synaptobrevin/VAMP family of R-type v-SNARE proteins
YDR468C	YGR009C	TLG1	SEC9	-	-	OG5_136368	fungi
YDR468C	YBR159W	TLG1	IFA38	TGME49_071890	5.20E-26	OG5_126812	short chain dehydrogenase.
YDR468C	YLL043W	TLG1	FPS1	-	-	OG5_126615	Plasma membrane channel, member of major intrinsic protein (MIP) family; involved in efflux of glycerol and in uptake of acetic acid and the trivalent metalloids arsenite and antimonite; phosphorylated by Hog1p MAPK under acetate stress, in plasmodium
YDR468C	YOR106W	TLG1	VAM3	-	-	OG5_139188	
YDR468C	YLR093C	TLG1	NYV1	-	-	OG5_147213	v-SNARE component of the vacuolar SNARE complex involved in vesicle fusion; inhibits ATP-dependent Ca(2+) transport activity of Pmc1p in the vacuolar membrane
YJL164C	YDR468C	TPK1	TLG1	TGME49_026030	2.80E-86	OG5_126748	cAMP dependent Kinase (AGC kinase)
YLL039C	YDR468C	UBI4	TLG1	TGME49_019820	1.10E-73	OG5_126703	Ubiquitin, becomes conjugated to proteins, marking them for selective degradation via the ubiquitin-26S proteasome system; essential for the cellular stress response; encoded as a polyubiquitin precursor comprised of 5 head-to-tail repeats
YGL095C	YDR468C	VPS45	TLG1	TGME49_071060	4.20E-70	OG5_127632	SM-protein
YKL196C	YDR468C	YKT6	TLG1	TGME49_015420	4.50E-34	OG5_127692	Vesicle membrane protein (v-SNARE) with acyltransferase activity; involved in trafficking to and within the Golgi, endocytic trafficking to the vacuole, and vacuolar fusion; membrane localization due to prenylation at the carboxy-terminus
YLR262C	YDR468C	YPT6	TLG1	TGME49_110460	1.50E-62	OG5_127692	Rab6

- Table S1 is associated with Figure 1 and shows a list of the putative interaction factors of TgStx1, and their function.
