# Supplementary material for: Toxoplasma gondii Syntaxin 6 Is Required for Vesicular Transport Between Endosomal-Like Compartments and the Golgi Complex
Source: Traffic. 2013 Sep 12;14(11):1166–81. doi: 10.1111/tra.12102 (PMC3963449; doi:10.1111/tra.12102)
Supplement: Table S2 — Primers used in this study. Restriction sites used for cloning are indicated. This table is associated with the Materials and Methods section and shows the primers used to construct the loxPStx6loxPYFP-HX vector. [file tra0014-1166-sd5.pdf]

Table S2. Primers used in this study

Primer Name	Sequence 5' - 3'	Restriction Site
Stx65'Rv	gtaCCTAGGcgccatgaattcTATAACTTCGTATAATGTATGCTATACG	AvrII
Stx65'UTRrv	cgcGAATTCtATAACTTCGTATAATGTATGCTATACGAAGTTATatggcgaccctgagaatggcac gaggctggg	EcoRI / LoxP
Stx63'UTRfw	gcgccGAGCTCccgtccaccggcctcggtctcccg	SacI
Stx63'UTRrv	gcgcgGAGCTCccgactcgctgctttccagacacc	SacI
